# Energy Landscape of State-Specific Electronic Structure Theory

**DOI:** 10.1021/acs.jctc.1c01089

**Published:** 2022-02-18

**Authors:** Hugh G. A. Burton

**Affiliations:** Physical and Theoretical Chemistry Laboratory, University of Oxford, South Parks Road, Oxford OX1 3QZ, United Kingdom

## Abstract

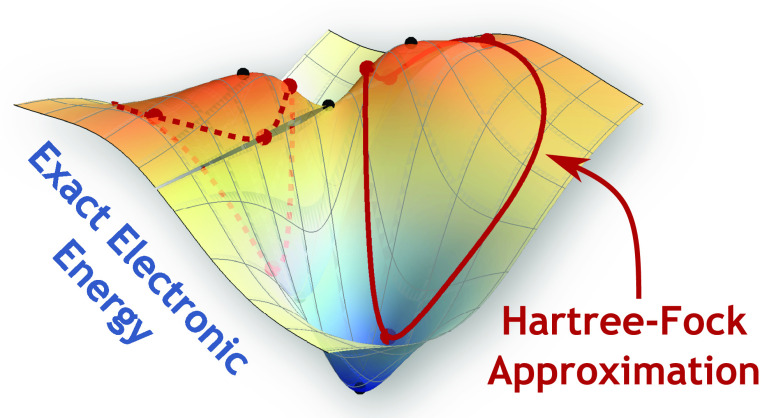

State-specific approximations can provide a more accurate representation of challenging electronic excitations by enabling relaxation of the electron density. While state-specific wave functions are known to be local minima or saddle points of the approximate energy, the global structure of the exact electronic energy remains largely unexplored. In this contribution, a geometric perspective on the exact electronic energy landscape is introduced. On the exact energy landscape, ground and excited states form stationary points constrained to the surface of a hypersphere, and the corresponding Hessian index increases at each excitation level. The connectivity between exact stationary points is investigated, and the square-magnitude of the exact energy gradient is shown to be directly proportional to the Hamiltonian variance. The minimal basis Hartree–Fock and excited-state mean-field representations of singlet H_2_ (STO-3G) are then used to explore how the exact energy landscape controls the existence and properties of state-specific approximations. In particular, approximate excited states correspond to constrained stationary points on the exact energy landscape, and their Hessian index also increases for higher energies. Finally, the properties of the exact energy are used to derive the structure of the variance optimization landscape and elucidate the challenges faced by variance optimization algorithms, including the presence of unphysical saddle points or maxima of the variance.

## I Introduction

Including wave function relaxation in state-specific approximations can provide an accurate representation of excited states where there is significant electron density rearrangement relative to the electronic ground state. This relaxation is particularly important for the description of charge transfer,^1−5^ core electron excitations,^6−9^ or Rydberg states with diffuse orbitals,^4,10,11^ and can be visualized using the eigenvectors of the difference density matrix for the excitation.^12,13^ In contrast, techniques based on linear response theory, including time-dependent Hartree–Fock^14^ (TD-HF), time-dependent density functional theory^15−17^ (TD-DFT), configuration interaction singles^16,18^ (CIS), and equation-of-motion coupled cluster theory^19,20^ (EOM-CC), are evaluated using the ground-state orbitals, making a balanced treatment of ground and excited states more difficult.^17^ Furthermore, linear response methods are generally applied under the adiabatic approximation and are limited to single excitations.^17,21^ In principle, state-specific approaches can approximate both single and double excitations,^1,22,23^ although the open-shell character of single excitations requires a multiconfigurational approach.^4,24−27^

Underpinning excited state-specific methods is the fundamental idea that ground-state wave functions can also be used to describe an electronic excited state. This philosophy relies on the existence of additional higher-energy mathematical solutions, which have been found in Hartree–Fock (HF),^22,28−48^ density functional theory (DFT),^49−52^ multiconfigurational self-consistent field (MC-SCF),^53−61^ and coupled cluster (CC) theory.^62−66^ These multiple solutions correspond to higher-energy stationary points of a parametrized approximate energy function, including local energy minima, saddle points, or maxima. It has long been known that the exact *k*th excited state forms a saddle point of the energy with *k* negative Hessian eigenvalues (where *k* = 0 is the ground state). These stationary properties have been identified using the exponential parametrization of MC-SCF calculations^53−56,67^ and can also be derived using local expansions around an exact eigenstate.^68,69^ However, questions remain about the global structure of the exact energy landscape and the connections between the exact excited states.

Multiple self-consistent field (SCF) solutions in HF or Kohn–Sham DFT are the most widely understood state-specific approximations. Their existence was first identified by Slater^28^ and later characterized in detail by Fukutome.^30−34^ Physically, these solutions appear to represent single-determinant approximations for excited states^1,22,70−72^ or mean-field quasi-diabatic states.^3,73^ In the presence of strong electron correlation, multiple SCF solutions often break symmetries of the exact Hamiltonian^38,41−43,46,47^ and can disappear as the molecular geometry changes.^35−37,45^ The stability analysis pioneered by Čižek and Paldus^74−77^ allows SCF solutions to be classified according to their Hessian index (the number of downhill orbital rotations). There are usually only a handful of low-energy SCF minima, connected by index-1 saddle points, while symmetry-broken solutions form several degenerate minima that are connected by higher-symmetry saddle points.^48^ At higher energies, stationary points representing excited states generally become higher-index saddle points of the energy.^48,50,78^

Recent interest in locating higher-energy SCF solutions has led to several new approaches, including modifying the iterative SCF approach with orbital occupation constraints^1,22^ or level-shifting;^10^ approximate second-order direct optimization of higher-energy stationary points;^79,80^ and minimizing an alternative functional such as the variance^4,27,81−83^ or the square-magnitude of the energy gradient.^52^ The success of these algorithms depends on the structure of the approximate energy landscape, the stationary properties of the excited states, and the quality of the initial guess. In principle, the approximate energy landscape is determined by the relationship between an approximate wave function and the exact energy landscape. However, the nature of this connection has not been widely investigated.

Beyond single-determinant methods, state-specific approximations using multiconfigurational wave functions have been developed to describe open-shell or statically correlated excited states, including MC-SCF,^53−56,60,61^ excited-state mean-field (ESMF) theory,^4,24−26^ half-projected HF,^81^ or multi-Slater–Jastrow functions.^83−85^ The additional complexity of these wave functions as compared to a single determinant has led to the use of direct second-order optimization algorithms^53−56,67^ or, more recently, methods based on variance optimization.^82,86,87^ Variance optimization exploits the fact that both ground and excited states form minima of the Hamiltonian variance ,^88^ and thus excited states can be identified using downhill minimization techniques. Alternatively, the folded-spectrum method uses an objective function with the form  to target the state with energy closest to ω.^89^ These approaches are particularly easy to combine with stochastic methods such as variational Monte Carlo^83,87,90^ (VMC) and have been proposed as an excited-state extension of variational quantum eigensolvers.^91,92^ However, variance optimization is prone to convergence issues that include drifting away from the intended target state,^23,83^ and very little is known about the properties of the variance optimization landscape or its stationary points.

In my opinion, our limited understanding about the relationship between exact and approximate state-specific solutions arises because exact electronic structure is traditionally viewed as a matrix eigenvalue problem, while state-specific approximations are considered as higher-energy stationary points of an energy landscape. The energy landscape concept is more familiar to theoretical chemists in the context of a molecular potential energy surface,^93^ where local minima correspond to stable atomic arrangements and index-1 saddles can be interpreted as reactive transition states.^94,95^ To bridge these concepts, this Article introduces a fully geometric perspective on exact electronic structure within a finite Hilbert space. In this representation, ground and excited states form stationary points of an energy landscape constrained to the surface of a unit hypersphere. Analyzing the differential geometry of this landscape reveals the stationary properties of ground and excited states and the pathways that connect them. Furthermore, the square-magnitude of the exact gradient is shown to be directly proportional to the Hamiltonian variance, allowing the structure of the exact variance optimization landscape to be derived. Finally, the relationship between approximate wave functions and the exact energy or variance is explored, revealing how the stationary properties of state-specific solutions are controlled by the structure of the exact energy landscape.

Throughout this work, key concepts are illustrated using the electronic singlet states of H_2_. While this minimal model is used to allow visualization of the exact energy landscape, the key conclusions are mathematically general and can be applied to any number of electrons or basis functions. Unless otherwise stated, all results are obtained with the STO-3G basis set^96^ using Mathematica 12.0^97^ and are available in an accompanying notebook available for download from 10.5281/zenodo.5615978. Atomic units are used throughout.

## II Exact Electronic Energy Landscape

### II.A Traditional Eigenvalue Representation

For a finite *N*-dimensional Hilbert space, the exact electronic wave function can be represented using a full configuration interaction (FCI) expansion constructed from a linear expansion of orthogonal Slater determinants as
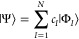
1This expansion is invariant to the particular choice of orthogonal Slater determinants |Φ_*I*_⟩, but the set of all excited configurations from a self-consistent HF determinant is most commonly used.^98^ Normalization of the wave function introduces a constraint on the expansion coefficients:
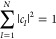
2while the electronic energy is given by the Hamiltonian expectation value:
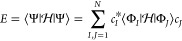
3

The optimal coefficients are conventionally identified by solving the secular equation:
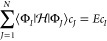
4giving the exact ground- and excited-state energies as the eigenvalues of the Hamiltonian matrix .

### II.B Differential Geometry of Electronic Structure Theory

In what follows, the wave function and Hamiltonian are assumed to be real-valued, although the key conclusions can be extended to complex wave functions. A geometric energy landscape for the exact electronic structure can be constructed by representing the wave function |Ψ⟩ as an *N*-dimensional vector  with coefficients:
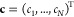
5

**Figure 1:**
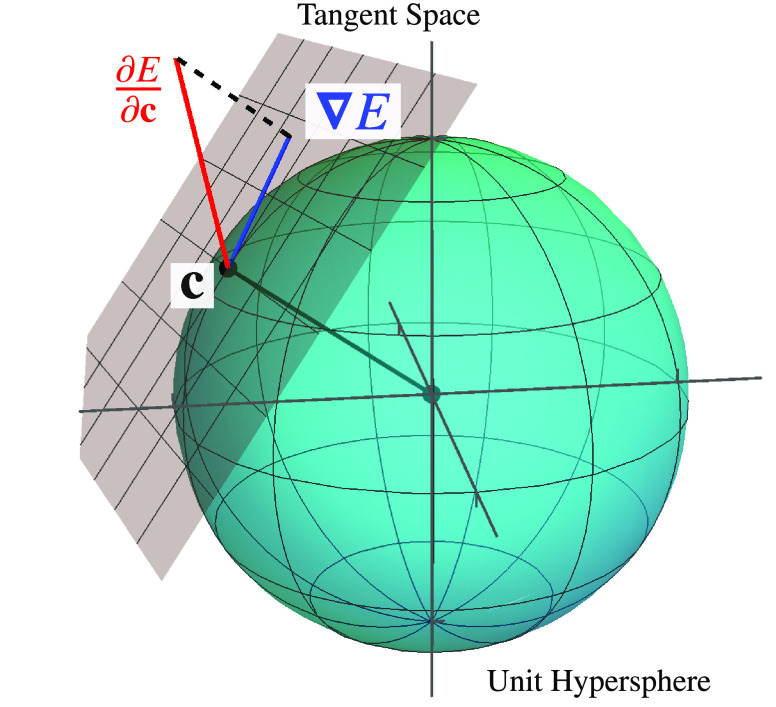
Geometric relationship between the position vector **c** (black) for a wave function constrained to the unit hypersphere, the global energy gradient in the full Hilbert space (red; eq 8), and the local gradient in the tangent space (blue; eq 12).

The energy is then defined by the quadratic expression:
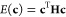
6where **H** represents the Hamiltonian matrix in the orthogonal basis of Slater determinants with elements .^98^ Normalization of the wave function is geometrically represented as
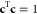
7and requires the coefficient vector **c** to be constrained to a unit hypersphere of dimension (*N* – 1) embedded in the full *N*-dimensional space (see Figure 1). In this representation, optimal ground and excited states are stationary points of the electronic energy constrained to the surface of this unit hypersphere.

The stationary conditions are obtained by applying the framework of differential geometry under orthogonality constraints, as described in ref (99). In particular, a stationary point requires that the global gradient of the energy in the full Hilbert space, given by the vector
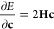
8has no component in the tangent space to the hypersphere. At a point **c** on the surface of the hypersphere, tangent vectors **Δ** satisfy the condition:^99^
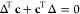
9The orthogonal basis vectors that span this (*N* – 1)-dimensional tangent space form the columns of a projector into the local tangent basis,^100^ denoted . Note that **c**_⊥_ forms a matrix whose columns span the tangent space, while **c** is a vector representing the current position. The corresponding projectors satisfy the completeness condition:
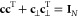
10where **I**_*N*_ is the *N*-dimensional identity matrix, and span disjoint vector spaces such that
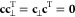
11The constrained energy gradient is then obtained by projecting the global gradient eq 8 into the tangent space to give
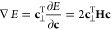
12with constrained stationary points satisfying ∇*E* = 0. Figure 1 illustrates this geometric relationship between the unit hypersphere, the exact tangent space, the global gradient in the full Hilbert space, and the local gradient in the tangent space.

The stationary condition ∇*E* = 0 requires that the global gradient eq 8 has no component in the tangent space. Therefore, it can only be satisfied if **Hc** is (anti)parallel to the position vector **c**. This condition immediately recovers the expected eigenvector expression **Hc**_*k*_ = *E*_*k*_**c**_*k*_, where the eigenvalue *E*_*k*_ is the exact energy of the *k*th excited state with coefficient vector **c**_*k*_. As a result, each exact eigenstate is represented by two stationary points on the hypersphere that are related by a sign-change in the wave function (i.e., ±**c**_*k*_ or, equivalently, ±|Ψ_*k*_⟩). There are no other stationary points on the exact landscape.

The exact hypersphere can be compared to the constraint surface in HF theory, where the occupied orbitals represent the current position on a Grassmann manifold and occupied–virtual orbital rotations define the tangent basis vectors.^99,101^ In HF theory, the global gradient is given by 2**FC**_occ_, where **F** is the Fock matrix and **C**_occ(vir)_ are the occupied (virtual) orbital coefficients. Projection into the Grassmann tangent space then yields the local gradient as 2**C**_vir_^T^**FC**_occ_, which corresponds to the virtual–occupied block of the Fock matrix in the molecular orbital (MO) basis.^48,101−103^ Therefore, in both HF theory and the exact formalism, optimization of the energy requires the off-diagonal blocks of an effective Hamiltonian matrix to become zero such that the “occupied–virtual” coupling between orbitals or many-particle states vanishes.

### II.C Properties of Exact Stationary Points

Stationary points on an energy landscape can be characterized as either minima, index-*k* saddles, or maxima, depending on the number of downhill directions (or negative eigenvalues of the Hessian matrix). Following ref (99), the analytic Hessian **Q** of the exact energy constrained to the hypersphere is given by
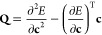
13

Taking the global second derivative in the full Hilbert space:
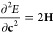
14and exploiting the relationship:
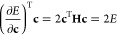
15allows the Hessian to be expressed as a shifted and rescaled Hamiltonian matrix:
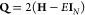
16Projecting into the space spanned by the tangent vectors then gives the constrained local Hessian as an (*N* – 1) × (*N* – 1) matrix defined as
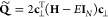
17where it should be remembered that **c**_⊥_ is an *N* × (*N* – 1) matrix. This expression for the Hessian of a CI wave function is also obtained with the exponential transformation used in second-order MC-SCF approaches.^55,104^

The constrained Hessian allows the properties of exact stationary points to be recovered. Consider a constrained stationary point **c**_*k*_ corresponding to an exact eigenstate |Ψ_*k*_⟩ with energy *E*_*k*_. To satisfy the completeness condition eq 10, the tangent basis vectors at this point must correspond to the remaining (*N* – 1) exact eigenstates. The local Hessian **Q̃** is then simply the full Hamiltonian shifted by *E*_*k*_ and projected into the basis of these (*N* – 1) exact eigenstates. As a result, the Hessian eigenvalues λ_*i*_ are directly proportional to the excitation energies, giving
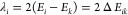
18where *E*_*i*_ are the exact energies with *i* ≠ *k*. Furthermore, the corresponding eigenvectors coincide with the position vectors **c**_*i*_ representing the remaining exact eigenstates |Ψ_*i*_⟩. Remarkably, this means that only the energy and second derivatives at an exact stationary point are required to deduce the electronic energies of the entire system. In other words, the full electronic energy spectrum is encoded in the local structure of the energy landscape around a single stationary point.

Now, at the stationary point **c**_*k*_ with energy *E*_*k*_, the number of exact eigenstates that are lower in energy is equal to the excitation level *k*. The number of negative eigenvalues λ_*i*_ = 2Δ*E*_*ik*_ is then equivalent to the excitation level. Significantly, there are only two minima on the exact energy landscape corresponding to positive and negative sign-permutations of the exact ground state, that is, ±|Ψ_0_⟩, and no higher-energy local minima. The *k*th excited state forms a pair of index-*k* saddle points that are also related by a sign-change in the wave function. The saddle-point nature of exact excited states was previously derived in the context of MC-SCF theory^53−56^ and has been described by Bacalis using local expansions around an excited state.^69^ In fact, the Hessian index has been suggested as a means of targeting and characterizing a particular MC-SCF excited state.^54,55^ In contrast, here the stationary properties of exact excited states have been derived using only the differential geometry of functions under orthogonality constraints. As will be shown later, this differential geometry also reveals the global structure of the energy landscape and the connections between exact eigenstates.

These properties also apply within a particular symmetry subspace. For example, the first excited state of a given symmetry is an index-1 saddle on the energy landscape projected into the corresponding symmetry subspace, but may be a higher-index saddle on the full energy landscape. For a pair of degenerate eigenstates, the corresponding stationary points will have a zero Hessian eigenvalue, and the corresponding eigenvector will interconvert the two states. Therefore, degenerate eigenstates form a flat continuum of stationary points on the exact energy landscape, and any linear combination of the two states must also be a stationary point of the energy.

**Figure 2:**
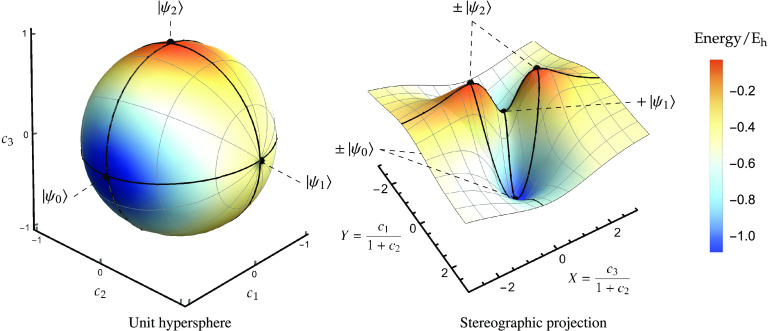
Left: Exact singlet electronic energy for H_2_ (STO-3G) at a bond length of 2 *a*_0_ constrained to the unit hypersphere. Right: Stereographic projection of the exact singlet energy using eq 20. Ground and excited states correspond to stationary points of the energy (black dots). The − |Ψ_1_⟩ state is at infinity in the stereographic representation. Each pair of stationary points is directly connected by a gradient extremal (black line) where the gradient is an eigenvector of the Hessian.

In Figure 2, the structure of the exact energy landscape is illustrated for the singlet states of H_2_ at a bond length of 2 *a*_0_ using the STO-3G basis set.^96^ An arbitrary spin-pure singlet wave function can be constructed as a linear combination of singlet configuration state functions to give
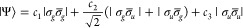
19Here, σ_*g*_ and σ_*u*_ are the symmetry-adapted MOs, and the absence (presence) of an overbar indicates an occupied high-spin (low-spin) orbital. A stereographic projection centered on (*c*_1_, *c*_2_, *c*_3_) = (0, 1, 0) is used to highlight the topology of the energy landscape (Figure 2, right panel), with new coordinates *X* and *Y* defined as
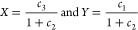
20The ground and excited states (black dots) form stationary points constrained to the hypersphere (Figure 2, left panel) with the global minima representing the ground state, index-1 saddles representing the first excited state, and the global maxima representing the second excited state (Figure 2, right panel). At the index-1 saddle, the downhill directions connect the two sign-permutations of the ground-state wave function, while the two uphill directions connect the sign-permutations of the second excited singlet state.

### II.D Gradient Extremals on the Electronic Energy Surface

Energy landscapes can also be characterized by the pathways that connect stationary points. For molecular potential energy surfaces, pathways can be interpreted as reaction trajectories between stable molecular structures, with saddle points representing reactive transition states.^95^ However, unlike stationary points, these pathways do not have a unique mathematical definition. On the exact electronic energy landscape, gradient extremals represent the most obvious pathways between stationary points. A gradient extremal is defined as a set of points where the gradient is either maximal or minimal along successive energy-constant contour lines.^105^ For the contour line with energy *E*_c_, these points can be identified by the constrained optimization:^98^
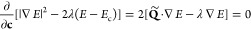
21Therefore, gradient extremals are pathways where the local gradient is an eigenvector of the local Hessian, that is:
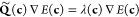
22These pathways propagate away from each stationary point along the “normal mode” eigenvectors of the Hessian and provide the softest or steepest ascents from a minimum.^105^

On the exact FCI landscape, gradient extremals directly connect each pair of stationary points, as illustrated by the black lines in Figure 2. Each extremal corresponds to the geodesic connecting the two stationary points along the surface of the hypersphere. The wave function along these pathways is only a linear combination of the two eigenstates at each end of the path. Therefore, gradient extremals provide a well-defined route along which a ground-state wave function can be continuously evolved into an excited-state wave function, or vice versa. Furthermore, as a gradient extremal moves from the lower-energy to the higher-energy stationary point, the corresponding Hessian eigenvalue λ(**c**) changes from positive to negative. This leads to an inflection point with λ(**c**) = 0 exactly halfway along each gradient extremal where the wave function is an equal combination of the two exact eigenstates. These inflection points are essential for understanding the structure of the variance optimization landscape in section II.E.

### II.E Structure of the Exact Variance Landscape

The accuracy of a general point on the exact energy landscape can be assessed using the square-magnitude of the local gradient, defined using eq 12 as
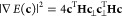
23Because all stationary points are minima of the squared-gradient with |∇*E*(**c**)|^2^ = 0, minimizing this objective function has been proposed as a way of locating higher-index saddle points in various contexts.^52,106,107^ For the electronic structure problem, exploiting the relationship between the tangent- and normal-space projectors (eq 10) allows eq 23 to be expressed using only the position vector **c** as
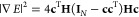
24Further expanding this expression gives
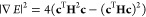
25which can be recognized as 4 times the Hamiltonian variance, that is:
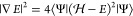
26While variance optimization has previously inspired the development of excited-state variational principles,^27,81−83,86,87^ these approaches have generally been motivated by the fact that exact eigenstates of  also have zero variance. In contrast, the relationship between the variance and |∇*E*|^2^ provides a purely geometric motivation behind searching for excited states in this way, derived from the structure of the exact energy landscape. Hait and Head-Gordon alluded to a relationship of this type by noticing similarities between the equations for SCF square-gradient minimization and optimizing the SCF variance.^52^

By connecting the squared-gradient of the energy to the variance, the exact energy landscape can be used to deduce the structure of the variance optimization landscape. Exact eigenstates form minima on the square-gradient landscape with |∇*E*|^2^ = 0. However, the square-gradient can have additional nonzero stationary points corresponding to local minima, higher-index saddle points, or local maxima.^108,109^ These “non-stationary” points do not represent stationary points of the energy, but they provide important information about the structure of the square-gradient landscape away from exact eigenstates. In particular, a stationary point of |∇*E*|^2^ with ∇*E* ≠ 0 can only occur when the local gradient is an eigenvector of the Hessian with a zero eigenvalue, **Q**(*c*)∇*E*(*c*) = 0.^108,109^ Nonstationary points therefore occur on the gradient extremals described in section II.D and correspond to the inflection points exactly halfway between each pair of eigenstates.

**Figure 3:**
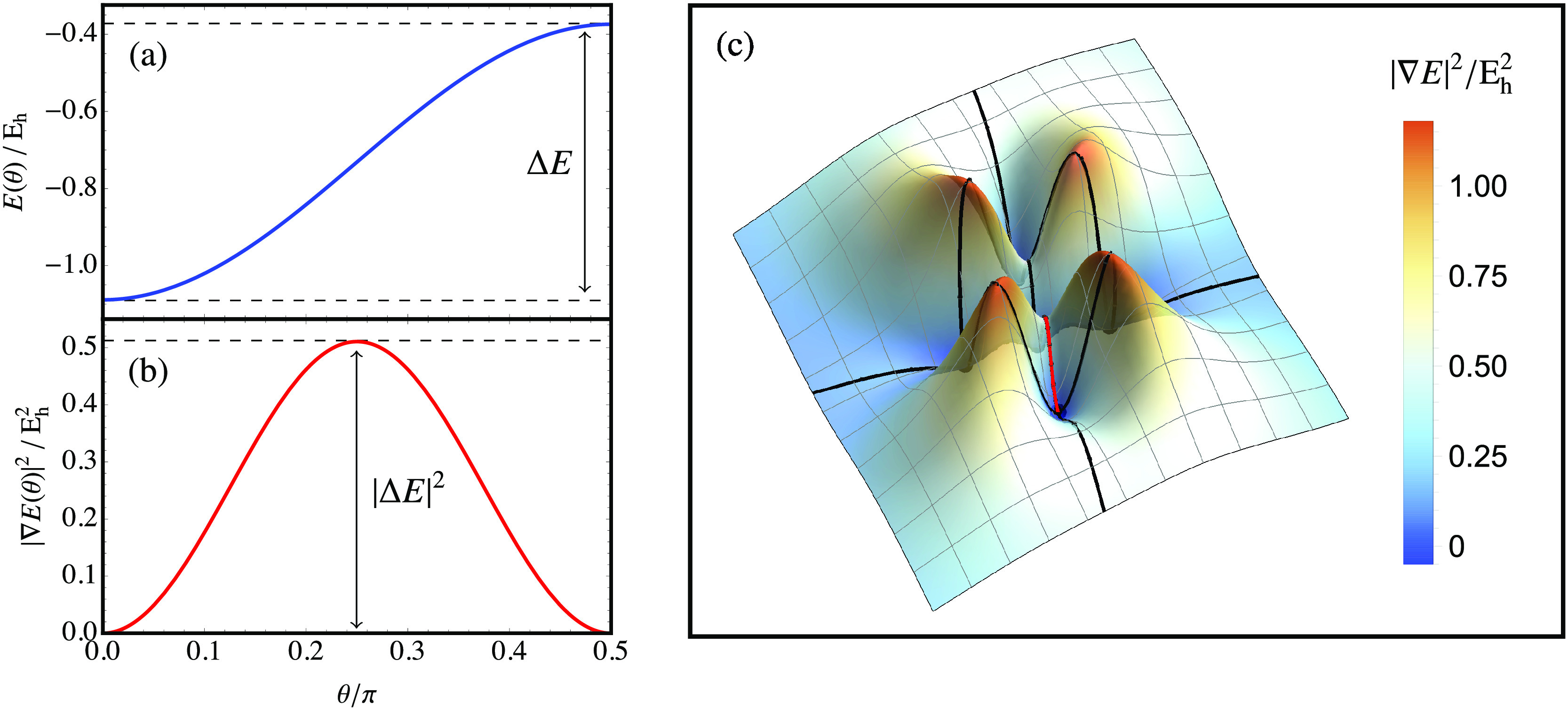
Energy square-gradient for the singlet states of H_2_ (STO-3G) at a bond length of 2 *a*_0_. (a) Energy along the gradient extremal connecting the ground and first excited singlet states. (b) Square-gradient of the energy along the gradient extremal connecting the ground and first excited singlet states. (c) Square-gradient landscape for singlet wave functions in H_2_ (STO-3G), with gradient extremals (black) connecting the physical minima. The gradient extremal plotted in (b) is highlighted in red.

Because gradient extremals only connect two eigenstates, the value of |∇*E*|^2^ at these nonstationary points can be obtained by parametrizing the wave function as
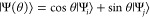
27The energy and square-gradient are then given by
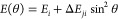
28a
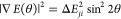
28bwhere 0 ≤ θ ≤ π/2 and Δ*E*_*ji*_ = *E*_*j*_ – *E*_*i*_. There are only two stationary points of the energy along each pathway, corresponding to |∇*E*|^2^ = 0 at θ = 0 and π/2, as illustrated for the gradient extremal connecting the ground and first excited singlet states of H_2_ (STO-3G) in Figure 3a. In contrast, the square-gradient has an additional stationary point at the inflection point θ = π/4 with |**∇***E*|^2^ = Δ*E*_*ji*_^2^ (see Figure 3b). This point corresponds to an unphysical maximum of |∇*E*|^2^ along the gradient extremal, and the height of this barrier depends on the square of the energy difference between the two states. Therefore, the exact square-gradient (or variance) landscape contains exact minima separated by higher-variance stationary points that form barriers at the inflection points of the energy, with the height of each barrier directly proportional to the square of the energy difference between the connected eigenstates. The lowest square-gradient barrier always connects states that are adjacent in energy to form an index-1 saddle point, while barriers connecting states that are not adjacent in energy form higher-index saddle points of |∇*E*|^2^. This structure of the exact square-gradient landscape is illustrated for the singlet states of H_2_ in Figure 3c.

Connecting the variance to the exact energy square-gradient reveals that the general structure of the variance optimization landscape is universal and completely determined by the energy difference between exact eigenstates. Systems with very small energy gaps will have low barriers between exact variance minima, while systems with well-separated energies will have high-variance barriers. This structure may also play a role in explaining the convergence behavior of variance minimization approaches, as discussed in section IV.

## III Understanding Approximate Wave Functions

### III.A Differential Geometry on the Exact Energy Landscape

Any normalized wave function approximation |Ψ̃(**t**)⟩ with variational parameters **t** can be represented as a point on the exact hypersphere using a linear expansion in the many-particle basis:
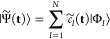
29Like the exact wave function, this expansion is invariant to the particular choice of orthogonal basis determinants. Because the number of parameters *t*_*i*_ is generally smaller than the Hilbert space size, the approximate wave functions form a constrained submanifold of the exact hypersphere. The structure of this approximate submanifold is implicitly defined by the mathematical form of the parametrization. Geometrically, the approximate energy is given as
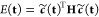
30and the constrained local gradient is defined as
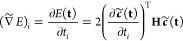
31Here, the partial derivatives of the coefficient vector define the local tangent basis of the approximate submanifold, representing the ket vectors:
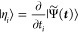
32In analogy with the exact wave function, the approximate local gradient corresponds to the global gradient (eq 8) projected into the space spanned by the approximate tangent vectors (eq 32). Optimal stationary points of the approximate energy then occur when **∇̃***E* = 0.

The HF approach illustrates how well-known approximate stationary conditions can be recovered with this geometric perspective. Although HF theory is usually presented as an iterative self-consistent approach,^110,111^ the HF wave function can also be parametrized using an exponential transformation of a reference determinant to give^76,102^
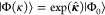
33Here, κ̂ is a unitary operator constructed from closed-shell single excitations and de-excitations, represented in second-quantization as^112^
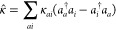
34where κ_*ai*_ are the variable parameters. The tangent vectors at **κ** = 0 correspond to the singly excited configurations, that is:
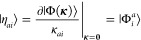
35These approximate tangent vectors span a subspace of the exact tangent space on the full hypersphere. Using eq 31, the local HF gradient is then given by^101,102^
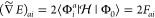
36which corresponds to twice the virtual–occupied components of the Fock matrix in the MO basis. As expected, the stationary condition **∇̃***E* = 0 recovers Brillouin’s theorem for HF convergence.^98^

### III.B Multiple Hartree–Fock Solutions

Although the HF wave function has fewer parameters than the exact wave function, there can be more HF stationary points than exact eigenstates.^29,45^ For example, in dissociated H_2_ with a minimal basis set, there are four closed-shell restricted HF (RHF) solutions and only three exact singlet states.^45^ In contrast to the exact eigenstates, there can also be multiple HF solutions with the same Hessian index, although this index generally increases with energy.^45^ Furthermore, HF solutions do not necessarily exist for all molecular geometries and can disappear at so-called “Coulson–Fischer” points.^28,40,45,46,48,113^ These phenomena can all be understood through the geometric mapping between the approximate HF submanifold and the exact energy landscape.

**Figure 4:**
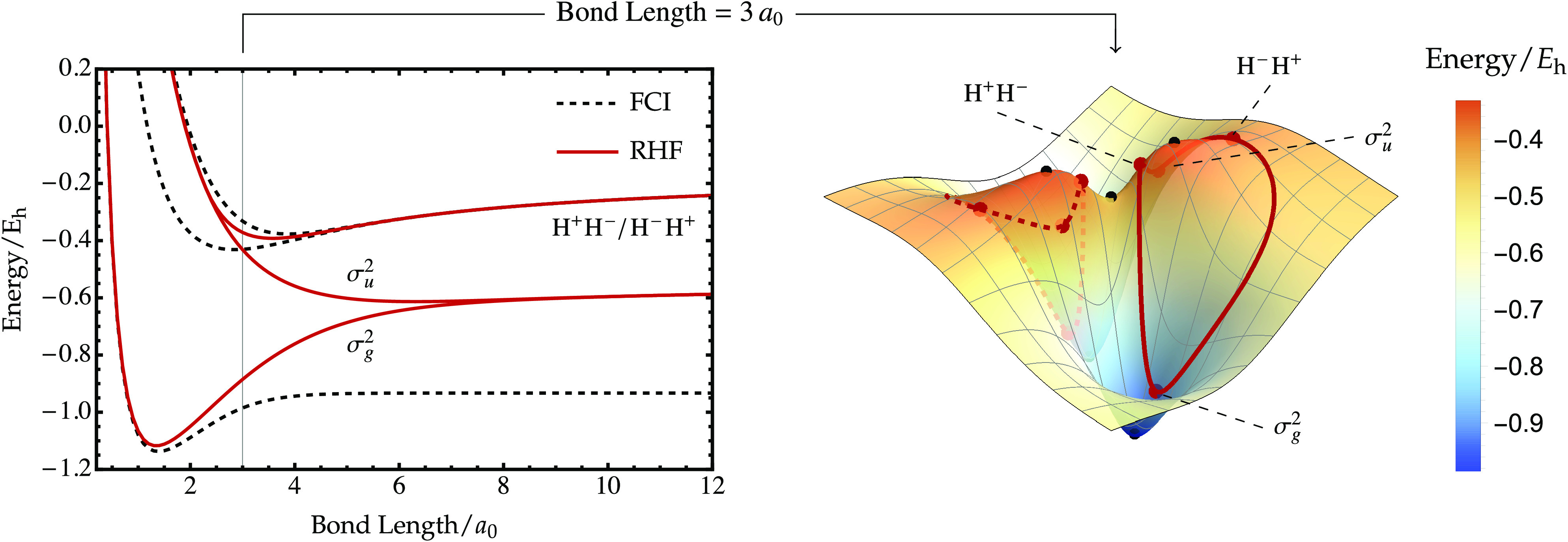
The space of possible RHF wave functions forms a one-dimensional submanifold (right panel; red curve) on the exact singlet energy surface for H_2_ (STO-3G) with a bond length of 3 *a*_0_. Multiple RHF solutions (left panel) correspond to constrained stationary points on the RHF manifold (right panel; red dots). The sign-permuted RHF wave functions are denoted by a dashed red curve (red panel).

Consider the RHF approximation for the singlet states of H_2_ (STO-3G). Only two RHF solutions exist at the equilibrium geometry, while an additional higher-energy pair of degenerate solutions emerge in the dissociation limit, as shown in the left panel of Figure 4 (see ref (45) for further details). In this system, the RHF submanifold forms a continuous one-dimensional subspace of the exact energy surface, illustrated by the red curve in Figure 4 (right panel). This submanifold includes all possible closed-shell Slater determinants for the system and is fixed by the wave function parametrization. Approximate solutions then correspond to constrained stationary points of the energy along the RHF submanifold, which occur when the exact energy gradient has no component parallel to the red curve. The existence and properties of these solutions are completely determined by the mapping between the RHF submanifold and the exact energy landscape.

**Figure 5:**
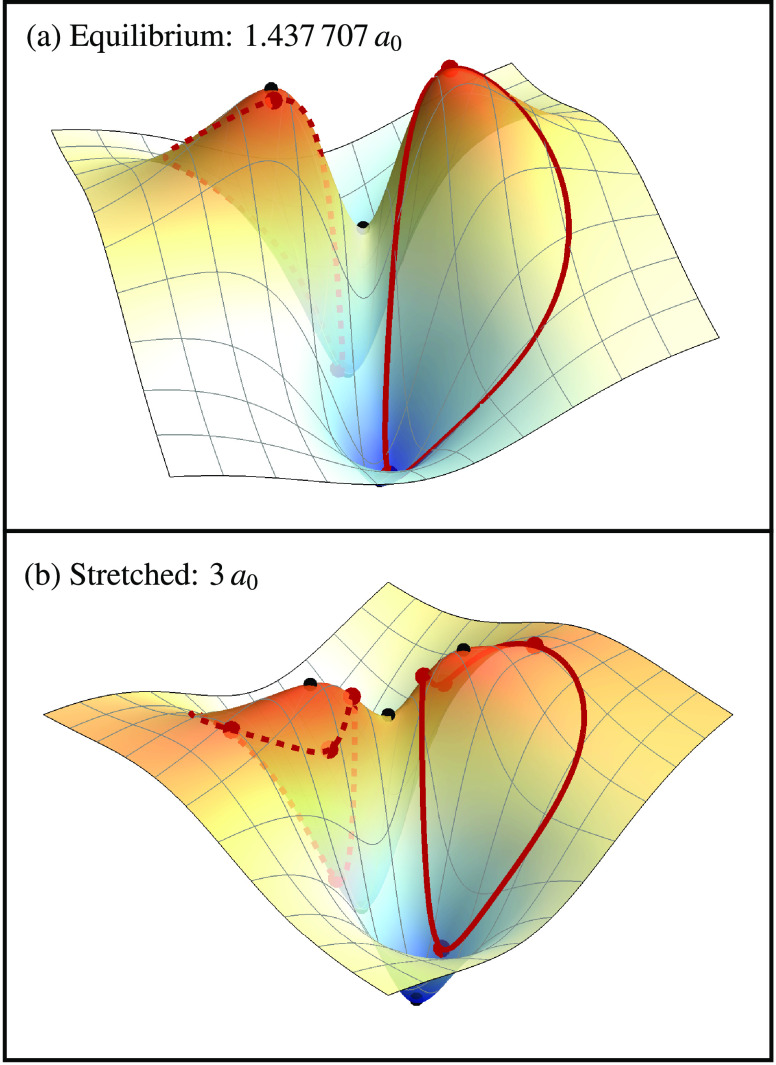
Mapping between the RHF submanifold (solid red line) and the exact singlet energy surface for H_2_ (STO-3G) at (a) the equilibrium bond length of 1.437707 *a*_0_ and (b) a stretched geometry of 3 *a*_0_. Sign-permuted RHF wave functions are denoted by dashed red curves.

At bond lengths near the equilibrium structure of H_2_ (STO-3G), the RHF submanifold extends relatively close to the exact global minimum and maximum, as illustrated in Figure 5a. This mapping results in only two constrained stationary points, the global minimum and maximum of the RHF energy, which correspond to the symmetry-pure σ_*g*_^2^ and σ_*u*_^2^ configurations, respectively. Notably, the RHF σ_*u*_^2^ global maximum represents a doubly excited state that cannot be accurately described by TD-HF or CIS.^17^ However, the RHF submanifold cannot get sufficiently close to the exact open-shell singlet state to provide a good approximation, and there is no stationary point representing this single excitation.

As the H–H bond is stretched toward dissociation, the exact energy landscape on the hypersphere changes while the RHF submanifold remains fixed by the functional form of the approximate wave function. Therefore, the exact energy evolves underneath the RHF submanifold, creating changes in the approximate energy that alter the properties of the constrained stationary points. For example, at a sufficiently large bond length, the RHF submanifold no longer provides an accurate approximation to the exact global maximum and instead encircles it to give two local maxima and a higher-energy local minimum of the RHF energy (Figure 5b). These local maxima represent the spatially symmetry-broken RHF solutions that tend toward to the ionic dissociation limit (left panel in Figure 4), while the higher-energy local minimum represents the σ_*u*_^2^ configuration. Combined with the global minimum, this gives a total of four RHF solutions, in contrast to only three exact singlet states. Consequently, we find that the number of RHF solutions can exceed the number of exact eigenstates because the RHF submanifold is a highly constrained nonlinear subspace of the exact energy landscape. Furthermore, it is the structure of this constrained subspace that creates a high-energy local minimum at dissociation, while the exact energy landscape has no local minima at any geometry.

**Figure 6:**
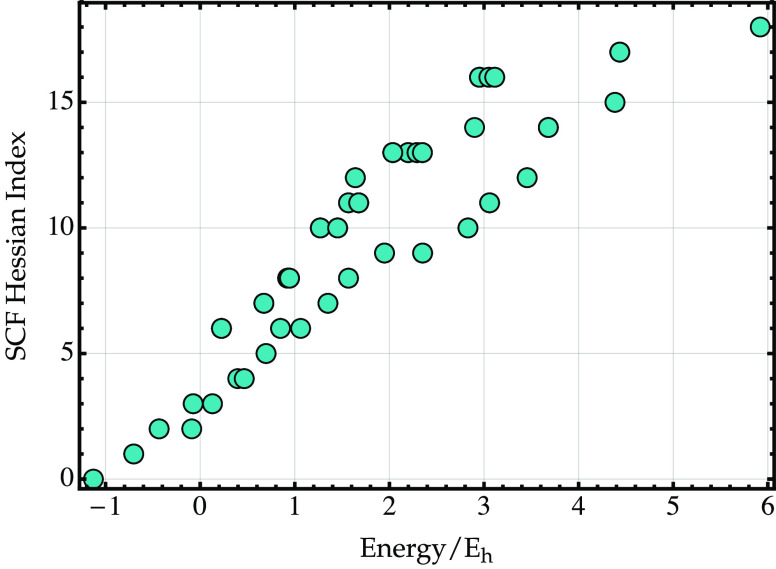
Comparison of the energy and SCF Hessian index computed for the orbital-optimized excited configurations of H_2_ (cc-pVDZ^114^) at *R* = 1.437707 *a*_0_ using unrestricted HF.

Finally, for real-valued HF wave functions, the Hessian of the approximate energy is computed using the second derivatives:^115^
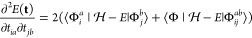
37where open-shell orbital rotations are now allowed. To investigate how the HF Hessian index changes with energy, the unrestricted excited configurations obtained from the ground-state RHF orbitals of H_2_ (cc-pVDZ^114^) at *R* = 1.437707 Å were optimized using the initial maximum overlap method^1^ in Q-Chem 5.4.^116^ The corresponding Hessian indices are plotted against the optimized energy in Figure 6. Similar to the exact eigenstates, there is a general increase in the Hessian index at higher energies. However, unlike the exact eigenstates, the approximate Hessian index does not increase monotonically with the energy. These results strengthen the conclusion of ref (48) that approximate HF excited states are generally higher-index saddle points of the energy.

### III.C Excited-State Mean-Field Theory

While multiple HF solutions are relatively well understood, the energy landscape of orbital-optimized post-HF wave functions remains less explored. The simplest excited-state extension of HF theory is the CIS wave function,^16,18^ constructed as a linear combination of singly excited determinants. CIS generally provides a qualitatively correct description of singly excited states, but it is less reliable for multiconfigurational or charge transfer excitations.^16^ The systematic overestimate of CIS for charge transfer excitations can be attributed to the absence of orbital relaxation effects.^117^ Therefore, to improve this description, the orbitals and CI coefficients can be simultaneously optimized to give the state-specific excited-state mean-field (ESMF) wave function, defined for singlet states as^4^
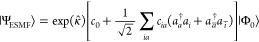
38Here, the reference determinant is retained in the expansion, and orbital rotations are parametrized by the unitary rotation defined in eq 34. In recent years, efficient optimization of this ansatz has been successfully applied to charge transfer and core excitations.^4,9,24,25^ Alternative approaches to include orbital relaxation effects in CIS using perturbative corrections have also been investigated.^2,118,119^

**Figure 7:**
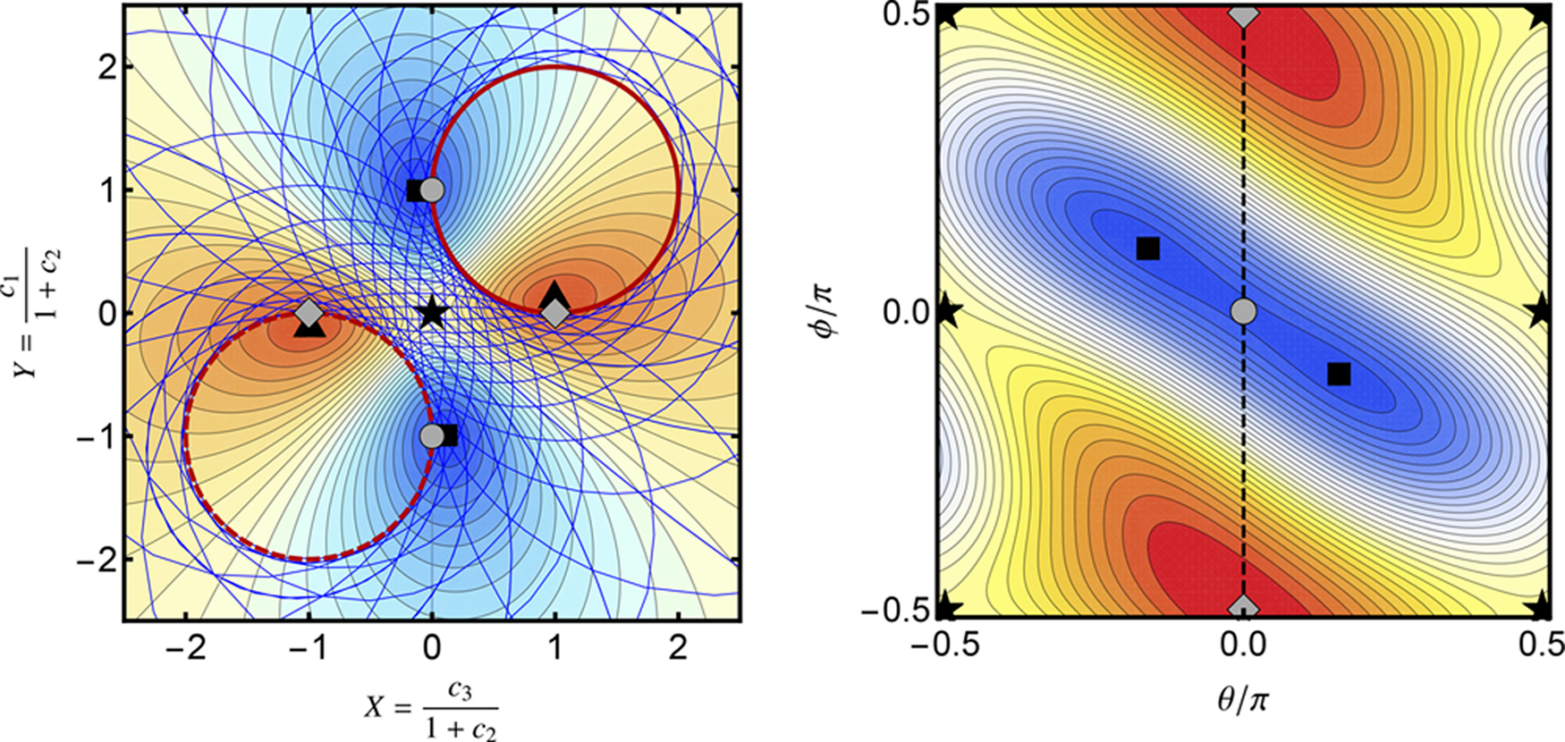
Left: RHF (solid and dashed red curves) and ESMF (blue mesh) constrained submanifolds superimposed on a stereographic projection (see eq 20) of the exact singlet energy landscape for H_2_ (STO-3G) at a bond length of *R* = 1.437707 *a*_0_. Exact stationary points and RHF solutions occur at the black and gray symbols, respectively. Right: The ESMF energy for singlet H_2_ (STO-3G) as a function of the wave function parameters (see eq 40). Black and gray symbols indicate stationary points of the ESMF or RHF energy, respectively, and the RHF submanifold corresponds to θ = 0 (dashed black line).

Geometrically, the single excitations for any closed-shell reference determinant define the tangent vectors to the RHF submanifold. Therefore, the orbital-optimized ESMF submanifold contains the RHF wave functions and all points that lie in the combined tangent spaces of the RHF submanifold, as illustrated for the singlet states of H_2_ (STO-3G) in Figure 7 (left panel). Like multiple RHF solutions, state-specific ESMF solutions correspond to stationary points of the energy constrained to the ESMF wave function manifold.

The ESMF wave function for singlet H_2_ (STO-3G) can be constructed by parametrizing the occupied and virtual molecular orbitals with a single rotation angle ϕ as
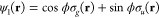
39a
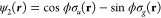
39band defining the normalized singlet CI expansion with a second rotation angle θ to give
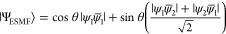
40The corresponding energy landscape at the equilibrium bond length *R* = 1.437707 *a*_0_ is shown in Figure 7 (right panel) as a function of θ and ϕ, with the RHF approximation indicated by the dashed black line at θ = 0. Stationary points representing the exact open-shell singlet state occur at  and , denoted by black stars, with optimal orbitals that correspond to σ_*g*_(**r**) and σ_*u*_(**r**). In common with the exact energy landscape, these open-shell singlet solutions form index-1 saddle points of the singlet energy. However, the local maximum representing the double excitation has no contribution from the single excitations and reduces to the closed-shell σ_*u*_^2^ RHF solution (gray ◆). This lack of improvement beyond RHF can be understood from the structure of the singlet ESMF submanifold: the exact double excitation is encircled by the RHF submanifold and therefore cannot be reached by any of the tangent spaces to the RHF wave function (Figure 7, left panel).

However, the most surprising observation from Figure 7 is not the higher-energy stationary points of the ESMF energy, but the location of the global minimum. Counterintuitively, the RHF ground state becomes an index-1 saddle point of the ESMF energy (gray ●), and there is a lower-energy solution at (θ, ϕ) = (±0.5026, ∓0.3304) that corresponds to the exact ground state (black ■). This lower-energy solution occurs because rotating the orbitals away from an optimal HF solution breaks Brillioun’s condition and introduces new coupling terms between the reference and singly excited configurations that allow the energy to be lowered below the RHF minimum. Because the RHF ground state is stationary with respect to both orbital rotations and the introduction of single excitations, this cooperative effect can only occur when the orbital and CI coefficients are optimized simultaneously. Furthermore, the combined orbital and CI Hessian is required to diagnose the RHF ground state as a saddle point of the ESMF energy, highlighting the importance of considering the full parametrized energy landscape.

The existence of an ESMF global minimum below the RHF ground state challenges the idea that CIS-based wave functions are only useful for approximating excited states. From a practical perspective, current ESMF calculations generally underestimate excitation energies because the multiconfigurational wave function used for the excited state can capture some electron correlation, while the single-determinant RHF ground state remains completely uncorrelated.^4^ Although CIS is often described as an uncorrelated excited-state theory, the wave function is inherently multiconfigurational and becomes correlated when the first-order density matrix is not idempotent.^120^ These circumstances generally correspond to excitations with more than one dominant natural transition orbital.^121^ Therefore, it is not too surprising that the ESMF global minimum can provide a correlated representation of the ground state. As a result, using state-specific ESMF wave functions for both the ground and the excited states should provide a more balanced description of an electronic excitation.

**Figure 8:**
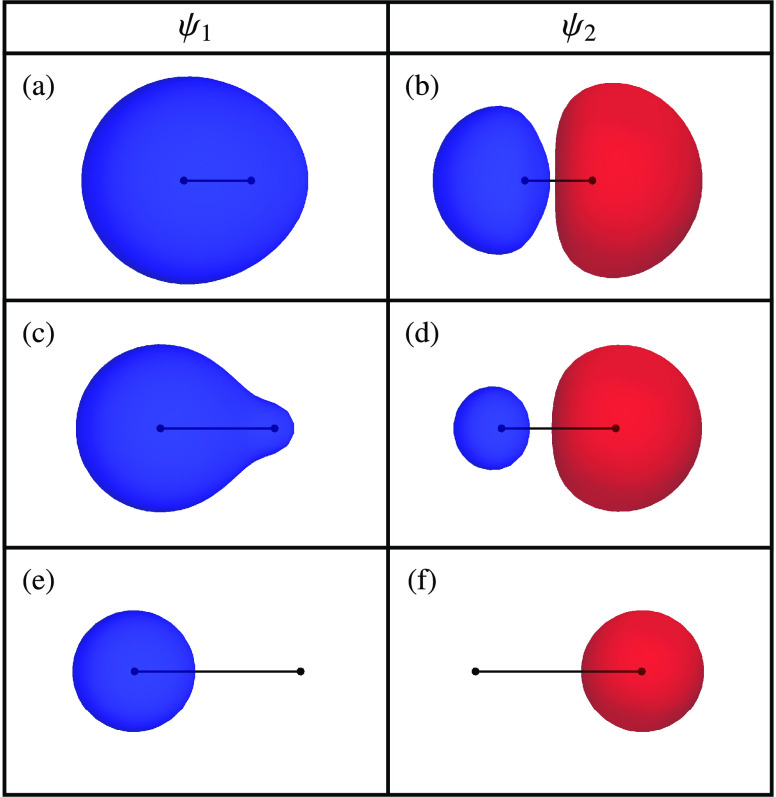
Spatial orbitals for the optimized ESMF ground state of H_2_ (STO-3G) at bond lengths of (a,b) 1.437707 *a*_0_, (c,d) 3.0 *a*_0_, and (e,f) 6.0 *a*_0_, plotted with an isosurface value of ±0.05. The reference configuration corresponds to |ψ_1_ψ̅_1_| with the ESMF wave function defined in eq 40.

Because the global minimum is exact across all H_2_ bond lengths, orbital-optimized ESMF may also provide an alternative reference wave function for capturing static correlation in single-bond breaking processes. The success of this ansatz can be compared to the spin-flip CIS (SF-CIS) approach, where the CI expansion is constructed using spin-flipping excitations from a high-spin reference determinant.^122^ SF-CIS gives an accurate description of the H_2_ ground state because single spin-flip excitations from an open-shell σ_*g*_σ_*u*_ reference produce both the σ_*g*_^2^ and the σ_*u*_^2^ configurations. In contrast, the ESMF global minimum contains a closed-shell reference determinant with symmetry-broken orbitals, as shown in Figure 8. These orbitals resemble the σ_*g*_ and σ_*u*_ MOs at short geometries (Figure 8a,b) where the reference determinant |ψ_1_ψ̅_1_| dominates the ESMF wave function. As the bond is stretched, the optimized orbitals localize on opposite H atoms to give an ionic reference state (Figure 8e,f), and the single excitations correspond to the localized configurations required for the diradical ground state.

### III.D Unphysical Solutions in Variance Optimization

Approximate state-specific variance minimization can also be considered as an optimization of the exact variance constrained to the approximate wave function submanifold. Variance minimization is increasingly being applied to target excited states because it turns the optimization of higher-energy stationary points into a minimization problem^27,81,82,86,87,123^ and is easily applied for correlated wave functions using VMC.^23,83^ In practice, these algorithms often use the folded-spectrum objective function:^89^
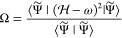
41to target an excited state with an energy near ω, before self-consistently updating ω until a variance stationary point is reached with ω = *E*.^27,81,83^ However, the structure of the variance landscape for approximate wave functions, and the properties of its stationary points, are relatively unexplored.

**Figure 9:**
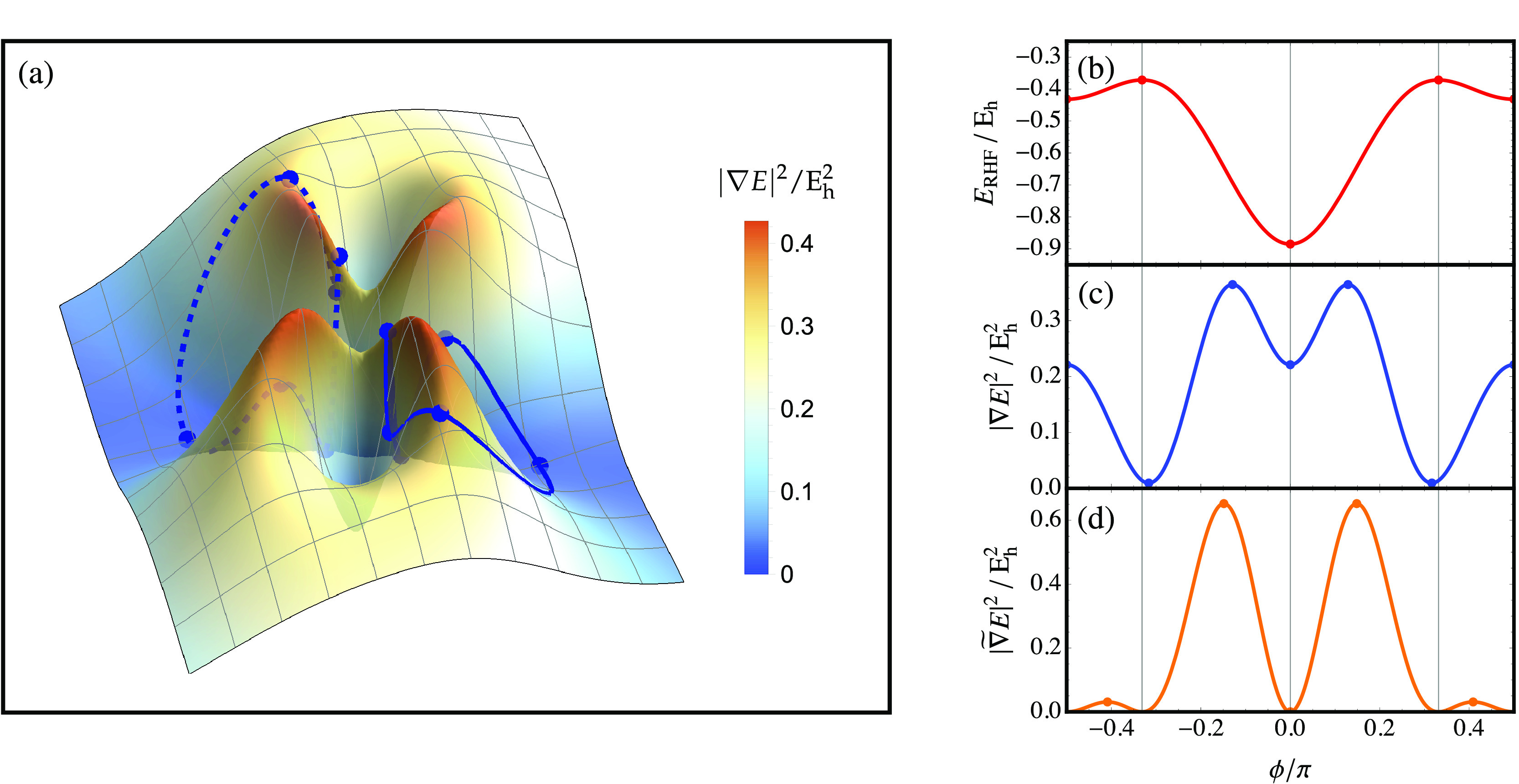
(a) Exact square-gradient landscape for singlet H_2_ (STO-3G) at a bond length of 3 *a*_0_. Restricted σ-SCF solutions identified using variance optimization are equivalent to constrained stationary points (blue dots) on the single-determinant subspace (blue curve) and its sign-related copy (dashed blue curve). (b) RHF energy as a function of the single orbital rotation angle ϕ (see eq 42). (c) Exact square-gradient of RHF wave functions with stationary points corresponding to σ-SCF solutions. (d) Square-magnitude of the constrained RHF energy gradient, with minima corresponding to RHF energy stationary points.

Because the variance is equivalent to the exact square-gradient |∇*E*|^2^, this landscape can be investigated using the mapping between an approximate wave function and the exact square-gradient landscape derived in section II.E. Consider the RHF approximation in H_2_ (STO-3G) with the doubly occupied orbital parametrized as
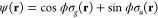
42Variance optimization for HF wave functions has been developed by Ye et al. as the iterative σ-SCF method, which uses a variance-based analogue of the Fock matrix.^27,81^ In Figure 9a, the RHF submanifold is shown as a subspace of the exact singlet square-gradient landscape at *R* = 3 *a*_0_, with optimal σ-SCF solutions corresponding to the constrained stationary points. Because the RHF approximation cannot reach any exact eigenstates in this system, the square-gradient is nonzero for all RHF wave functions, and there is no guarantee that the σ-SCF solutions coincide with stationary points of the constrained energy. This feature is illustrated in Figure 9b and c, where the energy and square-gradient are compared for the occupied orbital defined in eq 42. There are three constrained minima of the square-gradient for these RHF wave functions. The first at ϕ = 0 corresponds to the σ_*g*_^2^ RHF ground state, while the other two represent ionic configurations that are similar, but not identical, to the local maxima of the RHF energy.^27^ However, the σ_*u*_^2^ configuration, which forms a local minimum of the RHF energy, becomes a constrained local maximum of the energy square-gradient.

**Figure 10:**
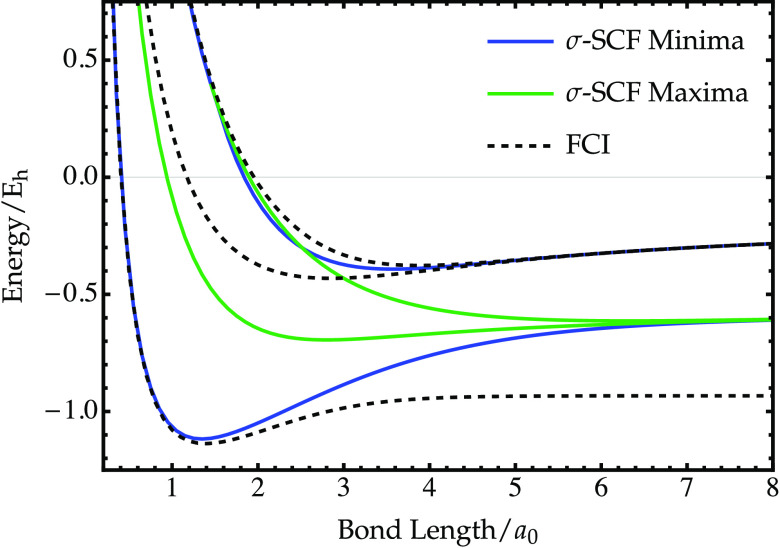
Energy of restricted σ-SCF solutions corresponding to constrained stationary points of the variance in H_2_ (STO-3G), including local minima (blue) and maxima (green). Exact singlet energies are shown for comparison (black dashed).

Energies corresponding to the complete set of RHF variance stationary points for H_2_ (STO-3G) are shown in Figure 10, with variance minima and maxima denoted by blue and green curves, respectively. These solutions closely mirror the low-energy σ-SCF states identified using the 3-21G basis set in ref (27). Despite becoming a local maximum of the variance at large bond lengths, the σ-SCF approach identifies the σ_*u*_^2^ solution at all geometries.^27^ Therefore, iterative methods such as σ-SCF must be capable of converging onto higher-index stationary points of the variance. However, not all higher-index stationary points correspond to physically meaningful solutions. For example, an additional degenerate pair of σ-SCF maxima can be found at all bond lengths in H_2_, with an energy that lies between that of the σ_*g*_^2^ and σ_*u*_^2^ solutions. These unphysical maxima exist where the RHF manifold passes over the square-gradient barriers created by nonstationary points on the exact energy landscape (see Figure 9a). The high nonconvexity of the exact square-gradient landscape means that these unphysical higher-index stationary points of the variance are likely to be very common for approximate wave functions.

As an alternative to variance minimization, excited state-specific wave functions can be identified by directly searching for points where the approximate local gradient becomes zero, **∇̃***E* = 0. For example, Shea and Neuscamman introduced an approach that modifies the objective function eq 41 using Lagrange multipliers to ensure that the approximate local gradient vanishes at a solution.^4^ Alternatively, Hait and Head-Gordon proposed the square-gradient minimization (SGM) algorithm that directly minimizes the square-magnitude of the local electronic gradient |**∇̃***E*^2^|.^52^ While the SGM approach is closely related to minimizing the exact variance, all stationary points of the approximate energy now become minima of local square-gradient with |**∇̃***E*|^2^ = 0, as shown for the RHF wave functions of H_2_ (STO-3G) in Figure 9d. Unphysical square-gradient stationary points, such as those resembling the RHF variance maxima in Figure 9c, can then be easily identified with |**∇̃***E*|^2^ ≠ 0. However, because the approximate energy can have more stationary points than the exact energy, there will be more nonstationary points of |**∇̃***E*|^2^ corresponding to inflection points between stationary states of the approximate energy (cf., Figure 9a and d). These additional nonstationary points will make the approximate |**∇̃***E*|^2^ landscape even more nonconvex than the constrained variance landscape, which can make numerical optimization increasingly difficult.

## IV Implications for Optimization Algorithms

We have seen that the exact energy landscape for real wave functions has only two minima, corresponding to negative and positive sign-permutations of the exact ground state. In addition, there is only one sign-permuted pair of index-*k* saddle points corresponding to the *k*th excited state. On the contrary, approximate methods may have higher-energy local minima and multiple index-*k* saddle points, although the maximum Hessian index is dictated by the number of approximate wave function parameters. These higher-energy local minima result from the wave function constraints introduced by lower-dimensional approximations.

The structure of the exact energy landscape highlights the importance of developing excited state-specific algorithms that can converge onto arbitrary saddle points of the energy. Any type of stationary point can be identified using iterative techniques that modify the SCF procedure, including the maximum overlap method^22,70^ and state-targeted energy projection.^10^ In addition, modified quasi-Newton optimization of the SCF energy^79,80^ should perform well, while state-specific CASSCF^60,61^ may also benefit from similar second-order optimization. Alternatively, modified eigenvector-following may allow excited states with a particular Hessian index to be targeted.^48,108,124^ However, methods that search for local minima of the energy, including SCF metadynamics^40^ and direct minimization,^101^ will perform less well for excited states and are better suited to locating the global minimum or symmetry-broken solutions.

In addition, the structure of the energy square-gradient landscape elucidates the challenges faced by variance optimization approaches. Each exact eigenstate forms a minimum of the variance, and minima adjacent in energy are separated by an index-1 saddle point with height proportional to the square of the corresponding energy difference.

Low barriers on the exact variance landscape offer a new perspective on the convergence drift observed in excited-state VMC calculations.^23,83^ In ref (83), variance optimization was found to drift away from the intended target state defined by the initial guess, passing through eigenstates sequentially in energy until converging onto the state with the lowest variance. By definition, a deterministic minimization algorithm cannot climb over a barrier to escape a variance minimum. However, the statistical uncertainty of stochastic VMC calculations means that they can climb a variance barrier if the height is sufficiently low. Essentially, the variance barrier becomes “hidden” by statistical noise. The index-1 variance saddle points connecting states adjacent in energy may then explain why the optimization drifts through eigenstates sequentially in energy^83^ and why this issue is more prevalent in systems with small energy gaps.^23^ Alternatively, the VMC wave function may simply not extend far enough into the exact variance basin of attraction of the target state to create an approximate local minimum. However, the use of highly sophisticated wave functions in ref (83) would suggest that this latter explanation is unlikely.

An additional concern is the presence of unphysical local variance minima or higher-index stationary points that occur when the exact variance is constrained to an approximate wave function manifold. For minimization algorithms such as VMC or generalized variational principles, the presence of many higher-index saddle points may increase the difficulty of convergence. There is also a risk of getting stuck in a spurious local minimum on the constrained manifold, which does not correspond to a physical minimum on the exact variance landscape. In contrast, iterative self-consistent algorithms such as σ-SCF^27,81^ are capable of converging onto higher-index stationary points, and it may be difficult to establish the physicality of these solutions. Therefore, iterative variance optimization requires careful analysis to ensure the physicality of solutions, for example, by using sufficiently accurate initial guesses.

Finally, although not considered in this work, the generalized variational principle developed by Neuscamman and co-workers includes Lagrange multipliers to simultaneously optimize multiple objective functions that target a particular excited eigenstate.^4,125,126^ The combined Lagrangian may include functionals that target a particular energy (such as eq 41), the square-magnitude of the local gradient, orthogonality to a nearby state, or a desirable dipole moment.^126^ This approach is particularly suited to systems where there is a good initial guess for the excited state or its properties, and a good choice of objective functions can significantly accelerate numerical convergence.^125^ These constraints may also help to prevent the drift of stochastic variance optimization algorithms by increasing the effective barrier heights between minima with the correct target properties.

## V Concluding Remarks

This contribution has introduced a geometric perspective on the energy landscape of exact and approximate state-specific electronic structure theory. In this framework, exact ground and excited states become stationary points of an energy landscape constrained to the surface of a unit hypersphere, while approximate wave functions form constrained subspaces. Furthermore, the Hamiltonian variance  is directly proportional to the square-magnitude of the exact energy gradient. Deriving this geometric framework allows exact and approximate excited state-specific methods to be investigated on an equal footing and leads to the following key results: (1) Exact excited states form saddle points of the energy with the number of downhill directions equal to the excitation level; (2) the local energy and second derivatives at an exact stationary point can be used to deduce the entire energy spectrum of a system; (3) the exact energy landscape has only two minima, corresponding to sign-permutations of the ground state; (4) approximate excited solutions are generally saddle points of the energy, and their Hessian index increases with the excitation energy; and (5) physical minima of the variance are separated from states adjacent in energy by index-1 saddle points, where the barrier height is proportional to the square of the energy difference between the two states.

While only the model H_2_ example has been considered, these results are sufficient to establish a set of guiding principles for developing robust optimization algorithms for state-specific excitations. Future work will investigate how Fermionic antisymmetry affects the relationship between exact and approximate electronic energy landscapes for systems with multiple same-spin electrons.

Beyond state-specific excitations, the exact energy landscape may also provide a new perspective for understanding the broader properties of wave function approximations. For example, this work has shown that the orbital-optimized ESMF wave function can describe the exact ground state of dissociated H_2_ (STO-3G) for all bond lengths using only the reference determinant and single excitations. This observation suggests that the orbital-optimized ESMF ground state may provide an alternative black-box wave function for capturing static correlation in single-bond dissociation. Alternatively, drawing analogies between a Taylor series approximation on the exact energy landscape and second-order perturbation theory may provide an orbital-free perspective on the divergence of perturbative methods for strongly correlated systems. Finally, investigating how more advanced methods such as multiconfigurational SCF,^104^ variational CC,^65,127^ or Jastrow-modified antisymmetric geminal power^128,129^ approximate exact stationary points on the energy landscape may inspire entirely new ground- and excited-state wave function approaches.
